# Anti‐Diabetic Effects of Ayanin, a Flavonoid Compound, in STZ/HFD‐Induced Diabetic Mice by Upregulating GLUT4 and Suppressing Macrophage‐Driven Inflammation in Adipose Tissues

**DOI:** 10.1002/fsn3.71429

**Published:** 2026-01-07

**Authors:** Yibing Lv, Chunyu Wu, Wenke Yang, Chenyang Wang, Jianmei Huang, Jinming Wang, XiaoLiang Xia, Dong Wu, Ke Yang, Zhenglong Guo, Shixiu Liao

**Affiliations:** ^1^ Henan Provincial Key Laboratory of Genetic Diseases and Functional Genomics, Medical Genetic Institute of Henan Provincial People's Hospital, People's Hospital of Zhengzhou University Zhengzhou China; ^2^ State Key Laboratory of Natural Medicines, School of Traditional Chinese Pharmacy China Pharmaceutical University Nanjing China

**Keywords:** AMPKα, Ayanin, diabetic, GLUT4, macrophages

## Abstract

Insulin resistance, marked by reduced GLUT4 expression in metabolic tissues such as adipose tissue and skeletal muscle, is a key contributor to the pathogenesis of Type 2 diabetes. Therapeutic agents that enhance GLUT4 expression in these tissues have demonstrated potential in the treatment of diabetes. Ayanin (AYN) is a flavonoid found in red wine grapes. This study evaluated the anti‐diabetic properties of AYN and investigated its mechanisms. In an in vivo study, the oral administration of AYN to diabetic mice resulted in ameliorated hyperglycemia and glucose tolerance and alleviated lipid dysfunction. The investigation into the underlying mechanisms revealed that AYN activated p‐AMPKα, which subsequently enhanced GLUT4 and CPT‐1α expression in both adipose and muscle tissues, as well as increased CPT‐1α expression in livers. In vitro experiments demonstrated that AYN activated AMPKα, increased GLUT4 expression, and facilitated glucose uptake in 3T3‐L1 adipocytes. Additionally, the metabolic dysfunctions related to lipids and glucose in adipocytes triggered inflammatory activation of macrophages within adipose tissue, leading to the exacerbation of insulin resistance and the downregulation of GLUT4 expression. AYN reduced macrophage infiltration and pro‐inflammatory mRNA expression in adipose tissues, contributing to the amelioration of diabetes. In vitro results for macrophages indicated that AYN decreased LPS‐induced pro‐inflammatory cytokine secretion. These findings suggest that AYN has potential for improving glucose tolerance and hyperglycemia, reducing insulin secretion, alleviating macrophage inflammation, and mitigating lipid dysfunction.

## Graphical abstract

Ayanin, a flavonoid extracted from red wine grapes, has demonstrated efficacy in ameliorating hyperglycemia, dyslipidemia, and insulin resistance through directly targeting the AMPKα/GLUT4 and AMPKα/CPT‐1α signaling pathways in metabolic tissues. This therapeutic effect is further augmented by Ayanin's capacity to attenuate macrophage‐induced inflammation within adipose tissues.

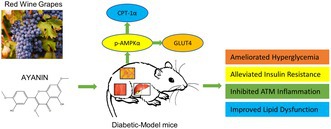

**Abbreviations**
AMPK: AMP‐activated protein kinase
AYN: Ayanin
epWAT: epididymal adipose tissue
HFD: high‐fat diet
LPS: lipopolysaccharide
SAT: subcutaneous adipose tissue
STZ: streptozocin
SVFs: stromal vascular fraction
T2DM: type 2 diabetes
WT: wild type

## 1 Introduction

Diabetes is one of the most prevalent chronic diseases globally, primarily characterized by hyperglycemia, impaired insulin secretion, and chronic inflammation (Esposito et al. 2024). In recent years, the incidence of diabetes has shown a steady increase, with projections indicating that the total number of diabetes cases will reach 700 million by 2045 (Saeedi et al. 2019). Over 90% of diabetic patients are diagnosed as having type 2 diabetes mellitus (T2DM), which is typically characterized by obesity, reduced insulin sensitivity, increased insulin secretion, and chronic inflammation in tissues (Lee and Olefsky 2021; Zheng et al. 2018). Chronic hyperglycemia and inflammation in T2DM patients lead to a range of complications, including cardiovascular and cerebrovascular diseases, cancer, kidney disease, and eye disorders (Demir et al. 2021; Esposito et al. 2024; Zheng et al. 2018). Although current clinical anti‐diabetic treatment regimens have demonstrated some efficacy, challenges such as drug‐induced side effects and resistance remain significant concerns (Grunberger 2017). Consequently, there is a pressing demand to develop safer and more effective anti‐diabetic therapies with minimal side effects (Lv et al. 2019).

China possesses a diverse array of edible natural resources, from which numerous potential hypoglycemic agents characterized by low toxicity, high efficacy, and safety have been identified (Kang et al. 2024). Previous studies have demonstrated that certain edible natural products, including okra, broccoli, and bitter gourd, exhibit significant hypoglycemic effects (Arigela et al. 2021; Geng et al. 2022; X. Li, Tian, et al. 2024). Within these commonly consumed foods, flavonoids such as apigenin and hesperidin have been identified as key components; however, their anti‐diabetic mechanisms remain inadequately understood (Allemailem et al. 2024; Mirzaei et al. 2023). Investigating the mechanisms and activities through which flavonoids ameliorate diabetic symptoms could establish a theoretical foundation for their use as complementary therapies in the dietary regimen of individuals with diabetes.

Ayanin (AYN) is a representative flavonoid compound predominantly found in edible plants, such as red wine grapes (Yu et al. 2024). It is currently identified that AYN exhibits a range of biological activities, including anti‐infection, anti‐allergic, and neuroprotective effects (Jin et al. 2023; Kawai et al. 2007; Yuan et al. 2017). Nonetheless, the potential anti‐diabetic properties of AYN and the underlying mechanisms remain unclear. In this study, it is proven that the administration of AYN ameliorated hyperglycemia and lipid dysfunction in diabetic mice induced by a high‐fat diet (HFD) in conjunction with streptozotocin (STZ). These findings suggest that AYN holds potential for ameliorating diabetes; however, the underlying mechanisms remain unclear.

GLUT4 (glucose transporter 4), predominantly expressed in adipose tissue and skeletal muscle, is a critical and well‐established target in the study of T2DM (Leto and Saltiel 2012). Its primary function is to facilitate glucose transport into cells, thereby reducing blood glucose levels. Reduced expression of GLUT4 in adipose tissue and skeletal muscle is a hallmark of insulin resistance and T2DM (Herman et al. 2022). Investigating agents that enhance GLUT4 expression in metabolic tissues may offer innovative and safe therapeutic options for T2DM (Lv et al. 2020). AYN‐treated adipose tissue and skeletal muscle in diabetic mice exhibited increased GLUT4 expression. Similarly, in vitro studies have demonstrated that AYN significantly upregulates GLUT4 expression in 3T3‐L1 adipocytes, thereby increasing glucose uptake. The activation of AMPKα signaling through phosphorylation at the Thr172 site is a well‐recognized mechanism for stimulating GLUT4 expression in metabolic tissues (Herzig and Shaw 2018). In this study, AYN was found to enhance GLUT4 expression by activating AMPKα signaling. Furthermore, AYN improved lipid metabolic dysfunction by upregulating CPT‐1α expression via AMPKα signaling.

Additionally, the development of T2DM is consistently associated with metabolic dysfunctions in adipose tissues (Ruze et al. 2023). The dysregulation of lipid and glucose metabolism in adipocytes triggers pro‐inflammatory activation of macrophages within adipose tissues (ATMs) (J. Li, Cai, et al. 2024), which subsequently inhibits the expression of GLUT4 in adipocytes, exacerbating insulin resistance (Oliver et al. 2010). Agents that inhibit the pro‐inflammatory activation of ATMs may help ameliorate insulin resistance by enhancing GLUT4 expression (Baek and Kim 2025). AYN treatment has been shown to inhibit the pro‐inflammatory activation of macrophages in both in vitro and in vivo studies.

In conclusion, AYN alleviates diabetic symptoms by activating the AMPKα/GLUT4 and AMPKα/CPT‐1α signaling pathway and reducing inflammation in adipose tissue macrophages, which in turn increases GLUT4 expression.

## 2 Materials and Methods

### 2.1 Chemicals and Regents

Ayanin (AYN) were purchased from Macklin company (A878393, endotoxin‐free, Shanghai, China). Minimum Essential Medium α (MEM‐α) were purchased from Hyclone (Logan, UT, USA). Fetal bovine serum (FBS) and antibiotics (100 U/mL penicillin and 100 μg/mL) were purchased from Sangon Biotech (Shanghai, China). The Insulin Elisa assay kits and FFA assay kit were provided by Jiancheng Bioengineering Institute (Nanjing, China). The blood glucose detector was purchased from Sinocare (Hunan, China). Antibodies of β‐actin, GLUT4, Cpt‐1α, AMPKα, p‐AMPKα and corresponding secondary antibodies were purchased from Proteintech Group (Wuhan, China). The enhanced chemiluminescence kits were provided by Servicebio company (Wuhan, China).

### 2.2 Animals and Treatments

All experiments involving animals were performed following the Guidelines for Animals Experiments of Zhengzhou University and were approved by the Animals Ethics Committee of Zhengzhou University (ZZUAFD2025‐0089). Male C57BL/6N mice, aged 8 weeks, were acquired from Beijing HFK Biosciences Co. Ltd. They were kept in a controlled setting with a 12‐h light/dark cycle at a temperature of 23°C ± 2°C and a humidity of 55% ± 10%, with unrestricted access to food and water. Following a week of adaptive feeding, the mice were initially split into two groups. The NC group (Normal control, *n* = 6) received a standard diet from Beijing HFK Bioscience Co. Ltd., while the remaining mice were given a HFD feeding (H10060, Beijing HFK Bioscience Co. Ltd) as an intervention group for inducing T2DM symptoms. For two consecutive days, mice in the intervention group received intraperitoneal injections of 40 mg/kg streptozotocin (BS185, Biosharp, China) citrate buffer solution (pH 4.5) following 4 weeks of a HFD feeding. Over the course of 2 weeks, the fasting blood glucose levels of the mice were assessed every 3 days. Mice with fasting blood levels higher than 11.1 mmol/L in the final test were classified as successful diabetic models. Randomly, the diabetic mice were divided into four groups: a group treated with a low dose of Ayanin (AYN, 30 mg/kg/day, *n* = 6), a group treated with a high dose of Ayanin (AYN, 60 mg/kg/day, *n* = 6), and a group treated with metformin (MET, 100 mg/kg/day, *n* = 6), and the vehicle control group (PBS, *n* = 6). Each day for 4 weeks, all mice received their agents orally. Weekly tests were conducted to measure the fasting blood glucose levels and body weight of the mice. On the 26th day of treatment, oral glucose tolerance (OGTT) and insulin tolerance test (ITT) were carried out on mice that had fasted for 12 h. A blood glucose detector (Sinocare, China) was used to obtain all data related to blood glucose levels.

### 2.3 Glucose Uptake Assay and Cell Culture

The 3T3‐L1 fibroblasts, provided by Servicebio company (Wuhan, China), were cultured in DMEM essential medium (SH30022.01, Hyclone, USA) containing 1% antibiotics (P7360, Solarbio, China) and 10% FBS (E510008, Sangon Biotech) in a humidified incubator at 37°C with ambient oxygen and 5% CO_2_. The 3T3‐L1 fibroblast differentiation into adipocytes was stimulated using different media containing insulin (10 mg/mL, HY‐P73243, MedChemExpress, China), dexamethasone (10 μM, HY‐14648, MedChemExpress, China), IBMX (0.5 M, HY‐12318, MedChemExpress, China), indomethacin (125 nM, HY‐14397, MedChemExpress, China), and rosiglitazone (1 μM, HY‐17386, MedChemExpress, China) for 5 days, followed by insulin (10 mg/mL) for 2 days. The glucose uptake level was determined with a Glucose Oxidase Method assay kit.

Peritoneal macrophages were collected from abdominal cavity lavage fluid of C57BL/6N mice, and macrophages were purified by adherence to plastic overnight (Qin et al. 2017). 10 ng/mL LPS (Lipopolysaccharide, HY‐D1056, MedChemExpress, China) was used to stimulate peritoneal macrophages, and AYN (116 μM) was used to antagonise the inflammatory activation of peritoneal macrophages induced by LPS. TNF‐α, IL‐6, and IL‐1β Elisa kit (Elabscience, Wuhan, China) were used to detect the concentration of peritoneal macrophages supernatants.

### 2.4 Histology, Immunohistochemistry and Biochemical Analysis

The techniques used for histology and immunohistochemistry were consistent with earlier studies (Lv et al. 2025). Hepatic steatosis in liver sections stained with hematoxylin and eosin (H&E) was assessed utilizing the hepatic steatosis scores. The grading scale was as follows: 0 indicating no steatosis, 1 representing 5% to 33% of hepatocytes exhibiting steatosis, 2 corresponding to 33% to 66% of hepatocytes with steatosis, and 3 denoting more than 66% of hepatocytes affected by steatosis (Kleiner et al. 2005). At the conclusion of the experiments, all mice were euthanized to obtain blood samples. The serum was separated and collected by centrifuging the blood samples at 3000 rpm for 15 min. The serum biochemical parameters, including total cholesterol (TC), triglycerides (TG), LDL‐C, and HDL‐C, were measured using an automatic biochemical analyzer (Hitachi 7180 + ISE, Tokyo, Japan). An assay kit from Nanjing Jiancheng Company (China) for free fatty acids (FFA) or insulin was employed to measure FFA and insulin concentrations. The levels of TC, TG, and FFA in liver and skeletal muscle were determined using assay kits from Nanjing Jiancheng Company (Nanjing, China).

### 2.5 Proteins Extracts and Western Blots

The extraction of proteins from 3T3‐L1 adipocytes, skeletal muscles, liver, and white adipose tissues followed a method reported earlier (Lv et al. 2020). In summary, the mature 3T3‐L1 adipocytes were treated for 12 h with either metformin, AYN (58 μM), AYN (116 μM), or a normal control (0.1% DMSO). Subsequently, cells were lysed using an ice‐cold RIPA buffer (50 mM Tris–HCl [pH 7.4], 150 mM NaCl, 1% NP‐40, 0.1% SDS) with a protease inhibitor cocktail (Roche, Basel, Switzerland) and a phosphatase inhibitor cocktail (Selleckchem, Houston, USA). The cell lysate was centrifuged at 15,000 rpm for 10 min to separate insoluble materials, and a BCA protein assay kit was employed to assess the protein concentration. Frozen samples of skeletal muscle, liver, and WAT were thawed to room temperature, weighed, pulverized, and then mixed with ice‐cold RIPA buffer containing both protease and phosphatase inhibitor cocktails. After cracking the mixture on ice for 30 min, it was centrifuged at 15,000 rpm for 15 min at 4°C. Subsequently, all insoluble material was discarded, and the protein concentration was measured using the BCA kit. According to previously outlined methods, proteins from tissues and cells were analyzed using Western blot.

### 2.6 Flow Cytometry

The stromal vascular fraction cells (SVFs) were collected according to previously established methods (Camell et al. 2017). The methods for cell staining were according to previous reports (Zhou et al. 2022). Briefly, collected SVFs from adipose tissues were blocked by CD16/CD32 antibodies and further stained by fluorescence‐labeled F4/80, CD45, CD11b, CD206, and CD11c antibodies for mice SVFs. FASC verse (BD Biosciences, San Jose, CA) and Flow jo software were used to collect and analyze the fluorescence of SVFs.

### 2.7 Real‐Time Quantitative Reverse Transcription PCR (RT‐qPCR)

Total RNA from epididymal adipose tissues (epWAT) was extracted and cDNA was synthesized. RT‐qPCR mixtures were prepared using a SYBR Green Real‐Time PCR kit (Bio‐rad, Hercules, CA, USA). mRNA levels were normalized to ACTB, and fold changes were determined using the 2^−ΔΔ*Ct*^ method. The Supporting Information include the primers for the detected mRNA.

### 2.8 Statistic and Analysis

A one‐way analysis of variance (ANOVA) was used to examine differences between groups. The data were presented as means ± standard error. Statistical analyses were conducted using Tukey's post hoc test in the GraphPad Prism 8.0 software. A *p* value < 0.05 was regarded as statistically significant.

## 3 Result

### 3.1 AYN Demonstrated Efficacy in Reducing Obese Body Weights, Blood Glucose Levels, and Impaired OGTT and ITT in Diabetic Mice

**FIGURE 1:**
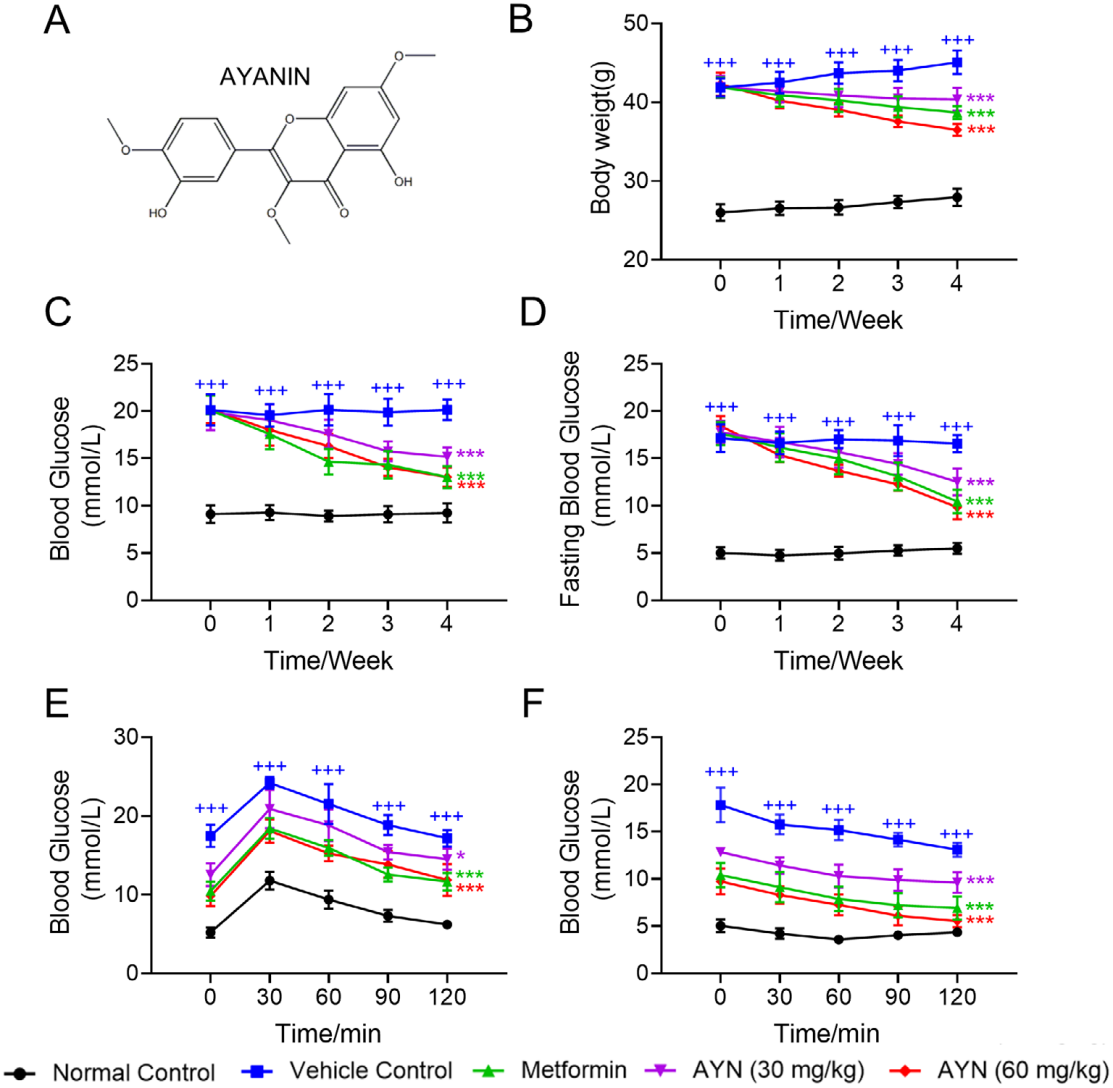
Anti‐diabetic effects of AYN in vivo. (A) Chemical structure of AYN; (B) AYN intervention decreased the body weight of diabetic mice; (C) AYN decreased the random blood glucose levels of diabetic mice; (D) AYN ameliorated fasting blood glucose levels of diabetic mice. (E, F) AYN ameliorated OGTT (E) and ITT (F) of diabetic mice. (*n* = 6, ^+++^ *p* < 0.001, compared with Normal control group; *p < 0.05, ***p < 0.001, compared with Vehicle control group).

To evaluate the anti‐diabetic effects of AYN, T2DM mice, induced by a HFD feeding in conjunction with STZ, were administered AYN orally. The metformin‐treated group served as the positive control, while the PBS‐treated group served as the vehicle control. Figure 1A showed the chemical structure of AYN. Prior to treatment, the body weight of mice in the vehicle control group was significantly higher than that of the normal control group (Figure 1B). Over the course of 4 weeks of AYN and metformin administration, the body weight of mice treated with AYN or metformin significantly decreased and was lower than that of the vehicle control group (Figure 1B). Additionally, 4 weeks of oral AYN administration led to a significant reduction in both random (Figure 1C) and fasting blood glucose levels (Figure 1D) in diabetic mice, exhibiting a dose‐dependent response. Notably, the group receiving 60 mg/kg AYN showed a substantial hypoglycemic effect compared to the metformin group (Figure 1C,D). In the study involving HFD and STZ induced T2DM mice, the diabetic mice exhibited impaired oral glucose tolerance (OGTT) and compromised insulin sensitivity (Lv et al. 2021). Following a four‐week treatment regimen with AYN at dosages of 30 and 60 mg/kg, it was observed that AYN ameliorated the impaired OGTT in these diabetic mice (Figure 1E). Notably, the group receiving 60 mg/kg of AYN demonstrated effects comparable to those observed in the metformin‐treated group. Specifically, in comparison to the diabetic model mice, those treated with AYN exhibited lower peak blood glucose levels and a more rapid decline post‐peak following glucose administration (Figure 1E). The insulin tolerance test (ITT), a critical indicator of insulin sensitivity in mice, revealed that in the group administered 60 mg/kg of AYN, blood glucose levels decreased rapidly and were significantly lower than those in the diabetic model group after intraperitoneal insulin injection. This finding indicates that a four‐week AYN treatment significantly enhanced insulin sensitivity in diabetic mice (Figure 1F). Overall, the results suggest that AYN effectively reduces blood glucose levels and body weight, enhances insulin sensitivity, and improves impaired oral glucose tolerance.

### 3.2 AYN Effectively Improved Excessive Insulin Secretion and Dyslipidemia in Diabetic Mice

**FIGURE 2:**
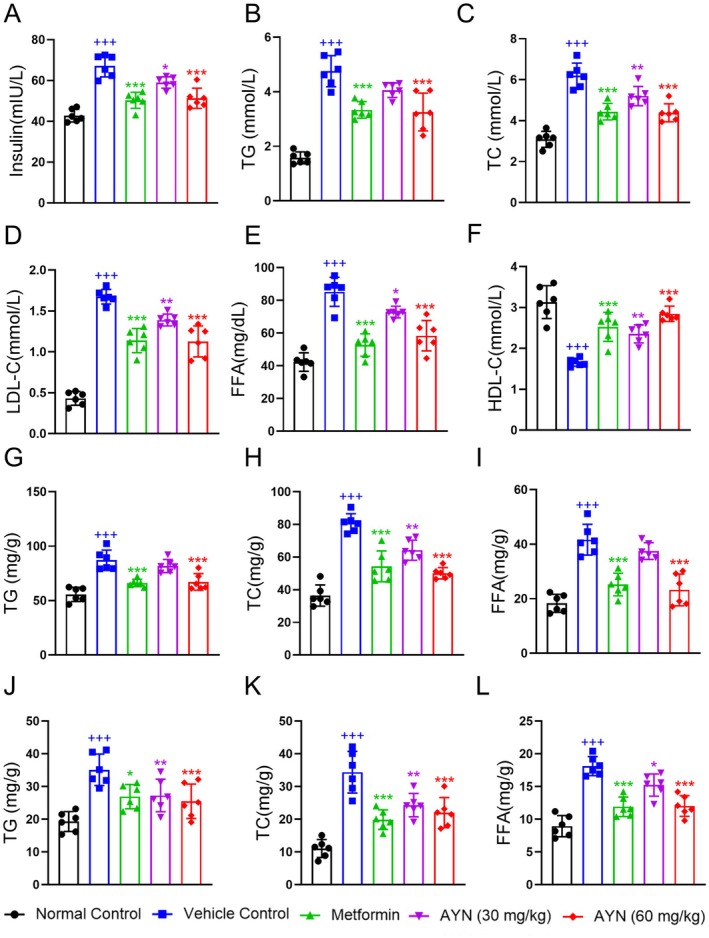
AYN decreased insulin concentration and ameliorated dyslipidemia of diabetic mice. (A) AYN decreased serum insulin concentration; AYN ameliorated abnormal TG (B), TC (C), LDL‐C (D), FFA (E) and HDL‐C (F) in serum of diabetic mice. (G–I) AYN administration decreased the TG (G), TC (H), and FFA (I) levels in livers of diabetic mice; (J–L) AYN administration decreased the TG (J), TC (K), and FFA (L) levels in skeletal muscles of diabetic mice. (*n* = 6, ^+++^ *p* < 0.001, compared with Normal control group; *p < 0.05, **p < 0.01, ***p < 0.001, compared with Vehicle control group).

The T2DM model, established through a low‐dose STZ injection in conjunction with prolonged HFD exposure, exhibited characteristics of insulin resistance accompanied by comparatively elevated insulin secretion (Lv et al. 2021). The findings demonstrated that serum insulin concentrations were significantly elevated in the vehicle group relative to the normal control group. Conversely, treatment with AYN and metformin resulted in a significant reduction in insulin concentrations, indicating that AYN effectively mitigated the excessive insulin secretion associated with insulin resistance (Figure 2A). A hallmark of T2DM is lipid metabolism disorder, which significantly contributes to insulin resistance (Gross et al. 2017). Serum lipid analysis in mice revealed that AYN administration significantly reduced levels of triglycerides (TG, Figure 2B), total cholesterol (TC, Figure 2C), low‐density lipoprotein cholesterol (LDL‐C, Figure 2D), and free fatty acids (FFA, Figure 2E), while significantly increasing high‐density lipoprotein cholesterol (HDL‐C, Figure 2F) levels. Additionally, our findings indicated that the administration of AYN led to a reduction of triglyceride, cholesterol, and free fatty acid levels in hepatic (Figure 2G–I) and muscular tissues (Figure 2J–L) to varying extents. In conclusion, these results suggest that AYN not only enhances glycemic control in diabetic mice but also mitigates lipid metabolism disorders associated with diabetes.

### 3.3 AYN Mitigates Hepatic Steatosis and Hypertrophic Adipocytes in Diabetic Mice

**FIGURE 3:**
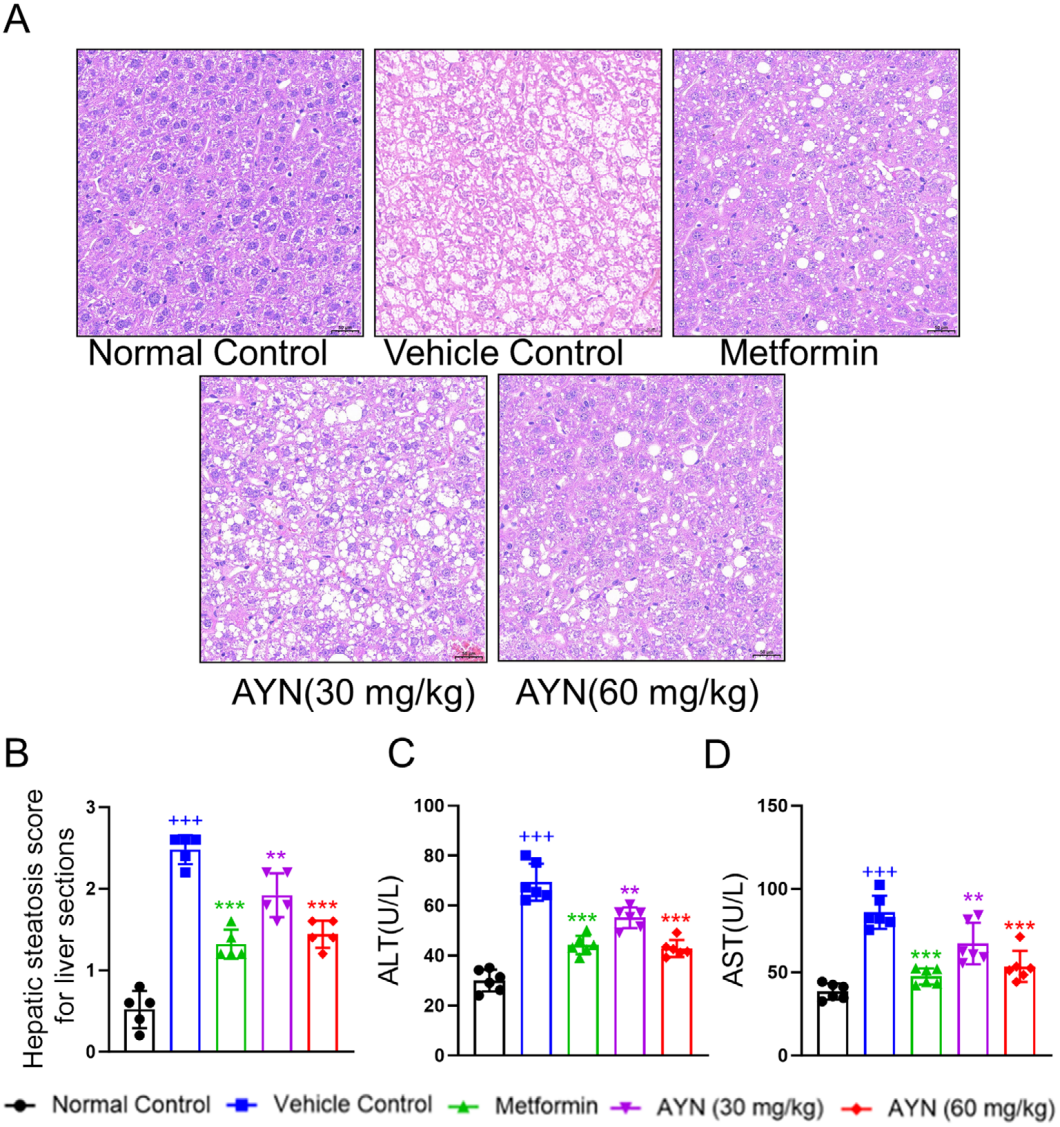
AYN ameliorated hepatic steatosis of diabetic mice. (A) AYN administration inhibited the liver vacuole symptoms of diabetic mice; (B) The mean hepatic steatosis scores for five slice fields were calculated for each group in the liver sections; (C, D) AYN administration decreased the ALT (C) and AST (D) of diabetic mice. (*n* = 6, ^+++^ *p* < 0.001, compared with Normal control group; *p < 0.05, **p < 0.01, ***p < 0.001, compared with Vehicle Control group).

The liver plays a pivotal role in glucose and lipid metabolism, and the onset of T2DM is frequently associated with excessive lipid accumulation in the liver, resulting in hepatic steatosis and reduced insulin sensitivity (Younossi et al. 2019). Histological analysis employing hematoxylin and eosin (HE) staining demonstrated that, after a four‐week regimen of AYN treatment, hepatic steatosis lesions in diabetic mice were markedly reduced (Figure 3A). Additionally, the hepatic steatosis scores of the livers in AYN‐treated mice showed a significant decrease (Figure 3B). In diabetic patients, chronic hepatic steatosis is associated with liver dysfunction, as evidenced by elevated levels of alanine aminotransferase (ALT) and aspartate aminotransferase (AST) (Younossi et al. 2019). In the vehicle‐treated group, diabetic mice exhibited increased ALT (Figure 3C) and AST (Figure 3D) levels compared to the normal control group, indicating impaired liver function. AYN administration resulted in a dose‐dependent reduction in ALT and AST levels, demonstrating that AYN effectively ameliorated both hepatic steatosis and liver dysfunction.

**FIGURE 4:**
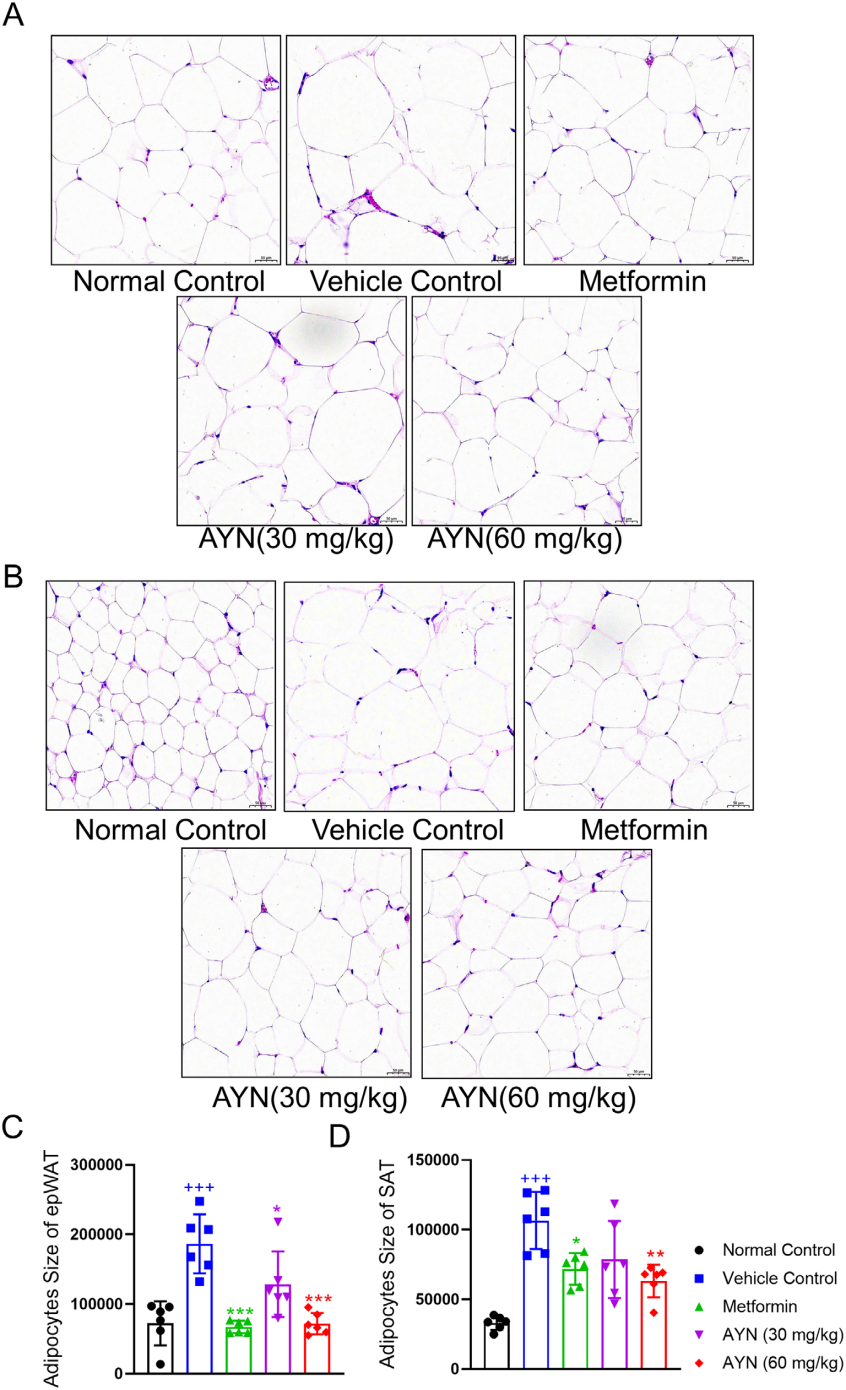
AYN ameliorated hypertrophic adipocytes of diabetic mice. (A) HE staining for epWAT; (B) HE staining for SAT; (C) Adipocyte areas of epWAT; (D) Adipocyte areas of SAT. (*n* = 6, ^+++^ *p* < 0.001, compared with Normal control group; *p < 0.05, **p < 0.01, ***p < 0.001, compared with Vehicle control group).

Adipose tissue serves as the primary site for lipid storage (Jeon et al. 2023). In individuals with T2DM, there is often an excessive accumulation of lipids within adipocytes in adipose tissue, leading to pathological hypertrophy and irregular morphological alterations (Ahmed et al. 2021). HE staining of epididymal white adipose tissue (epWAT) (Figure 4A,C) and subcutaneous adipose tissue (SAT) (Figure 4B,D) revealed that adipocytes in the vehicle control group displayed irregular shapes and increased volumes. In contrast, the group receiving AYN treatment exhibited a significant reduction in adipocyte volume and normalization of adipocyte morphology (Figure 4A–D). These observations indicate that AYN administration may ameliorate the metabolic microenvironment of adipose tissue and mitigate adipocyte hypertrophy.

### 3.4 AYN Mitigates T2DM Through the Activation of the AMPKα Signaling and the Upregulation of GLUT4 and CPT1α Expression

**FIGURE 5:**
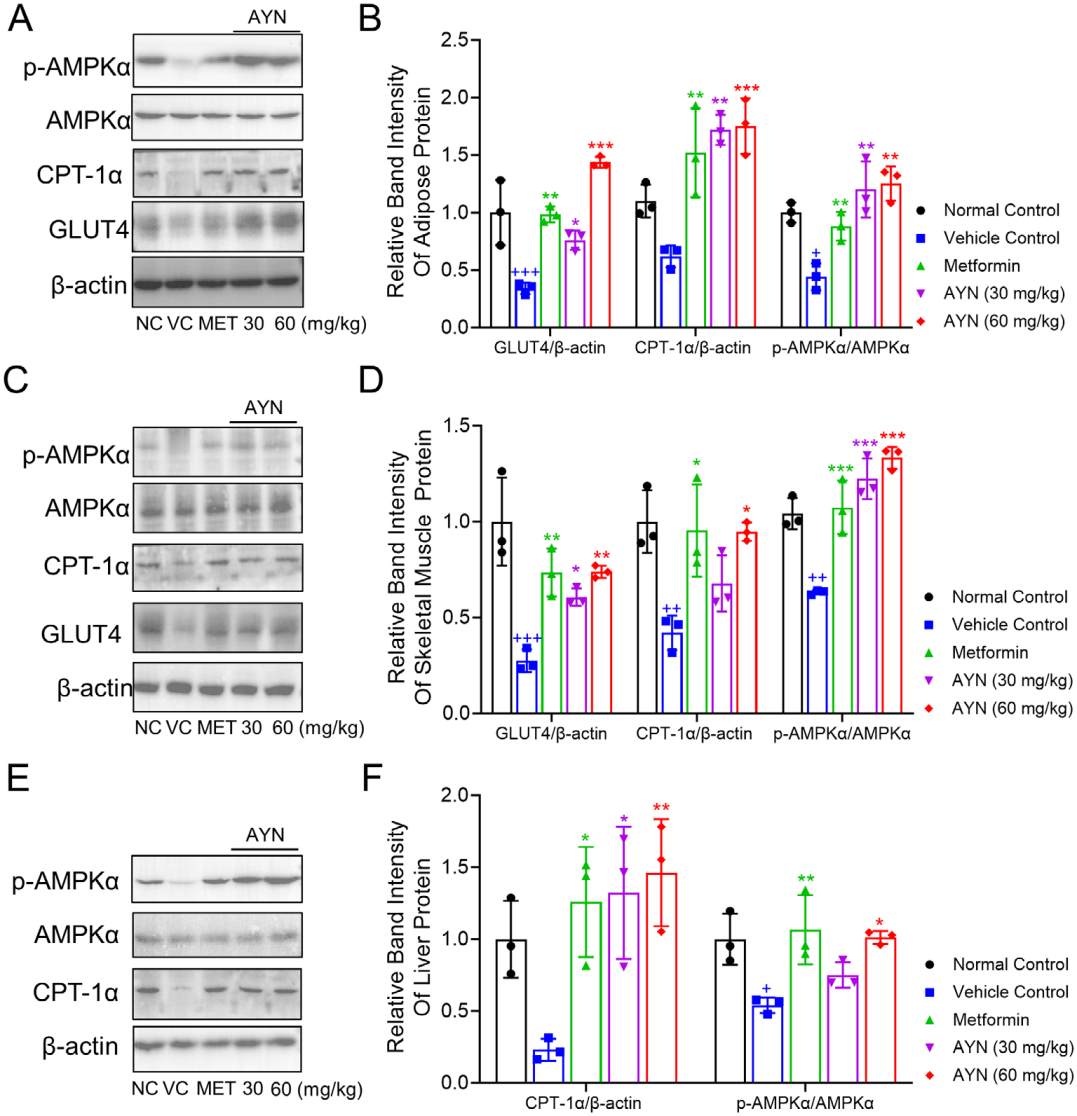
AYN activated AMPKα pathway in tissues. (A) AYN intervention increased p‐AMPKα, GLUT4, and CPT‐1α expression in epWAT; (B) Relative band intensity for GLUT4/β‐Actin, CPT‐1α/β‐Actin, p‐AMPKα/AMPKα for protein in epWAT; (C) AYN intervention increased p‐AMPKα, GLUT4, and CPT‐1α expression in skeletal muscles; (D) Relative band intensity for GLUT4/β‐Actin, CPT‐1α/β‐Actin, p‐AMPKα/AMPKα for protein in skeletal muscles. (E) AYN intervention increased p‐AMPKα and CPT‐1α expression in livers; (F) Relative band intensity for CPT‐1α/β‐Actin, p‐AMPKα/AMPKα for protein in livers. (*n* = 3, ^+++^ *p* < 0.001, compared with Normal control group; *p < 0.05, **p < 0.01, ***p < 0.001, compared with Vehicle control group).

GLUT4 is a critical glucose transporter protein that facilitates the uptake of glucose from the extracellular environment into cells, such as those in muscle and adipose tissues, in response to insulin, thereby enhancing glucose metabolism and utilization (Leto and Saltiel 2012; Watson and Pessin 2006). Flavonoids have been documented to show potentials on augmenting GLUT4 expression and improving glucose uptake (Lv et al. 2021). To evaluate whether AYN contributes to diabetes management by enhancing GLUT4 expression, the expression levels of GLUT4 in the adipose and skeletal muscle tissues of mice were analyzed using western blot analysis. Following a four‐week administration of AYN, a significant upregulation of GLUT4 expression was observed in the epWAT (Figure 5A,B) and skeletal muscle tissues (Figure 5C,D) of diabetic mice treated with AYN, with the most pronounced effect noted in the group receiving 60 mg/kg of AYN. AMPKα signaling functions as a pivotal regulatory mechanism for cellular energy homeostasis, with phosphorylation at the Thr172 site of AMPKα facilitating the expression of GLUT4 (Wu et al. 2024). To investigate whether AYN augments GLUT4 expression through activation of the AMPKα signaling pathway, the expression levels of phosphorylated AMPKα (p‐AMPKα) in various tissues were assessed. The findings demonstrated that both 30 and 60 mg/kg AYN increased p‐AMPKα expression, indicating that AYN may enhance GLUT4 expression via activation of the AMPKα signaling pathway, thereby improving glucose uptake in adipose (Figure 5A,B) and muscle tissues (Figure 5C,D). Furthermore, CPT‐1α, a critical rate‐limiting enzyme involved in fatty acid β‐oxidation and regulated by AMPKα, also contributes to the regulation of triglyceride and lipid synthesis (Dong et al. 2024). The upregulation of CPT‐1α expression promotes lipid metabolism, reduces lipid accumulation in tissues, and mitigates hepatic steatosis and adipocyte hypertrophy (Dong et al. 2024; Fondevila et al. 2022). Administration of AYN significantly increased CPT‐1α expression in the epWAT (Figure 5A,B), skeletal muscle (Figure 5C,D), and liver (Figure 5E,F), which was accompanied by elevated levels of p‐AMPKα expression. This suggests that AYN may alleviate lipid metabolism disorders associated with diabetes by activating the AMPKα/CPT‐1α signaling pathway.

### 3.5 AYN Facilitated the Expression of GLUT4 in 3T3‐L1 Cells Through the Activation of the AMPKα Signaling Pathway

**FIGURE 6:**
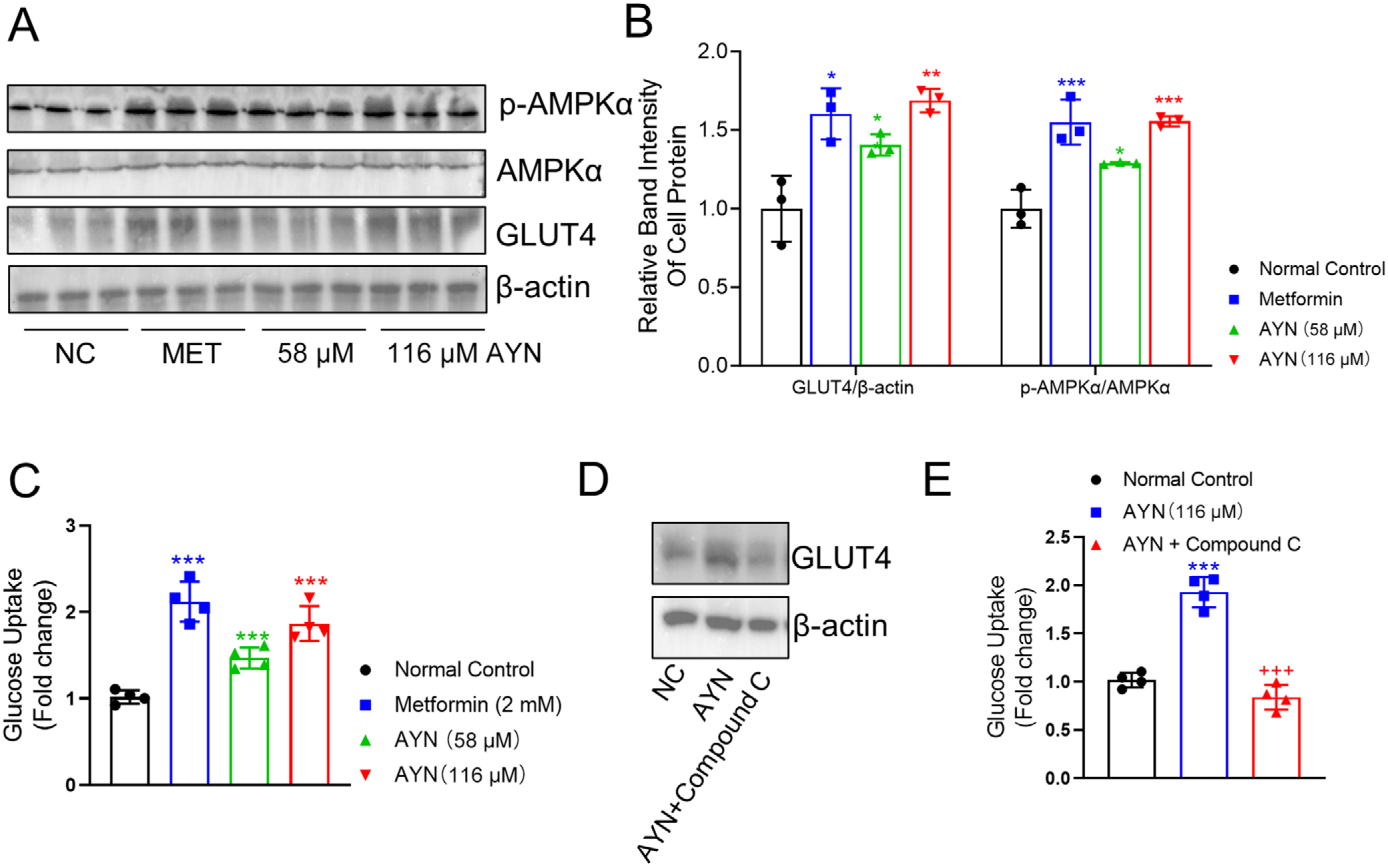
AYN increased glucose uptake of 3T3‐L1 adipocytes by activating AMPKα/GLUT4 pathway. (A) AYN increased GLUT4 and p‐AMPKα expression in 3T3‐L1 adipocytes; (B) Relative band intensity for GLUT4/β‐Actin, p‐AMPKα/AMPKα for protein in 3T3‐L1 adipocytes; (C) AYN increased the glucose uptake of 3T3‐L1 adipocytes; (D) AMPKα inhibitor, Compound C, suppressed the GLUT4 expression of 3T3‐L1 adipocytes induced by AYN; (E) Compound C inhibited the glucose uptake of 3T3‐L1 adipocytes induced by AYN. (*n* = 3, *p < 0.05, **p < 0.01, ***p < 0.001, compared with Normal control group; ^+++^ *p* < 0.001, compared with AYN group in E).

In vitro experiments involved stimulating 3T3‐L1 adipocytes with two distinct concentrations of AYN, followed by protein extraction for subsequent analysis. Consistent with in vivo protein detection results, AYN was found to enhance the expression of p‐AMPKα and GLUT4 in 3T3‐L1 adipocytes, exhibiting a concentration‐dependent effect (Figure 6A,B). With the increased expression of GLUT4, AYN significantly augmented glucose uptake in 3T3‐L1 adipocytes (Figure 6C). Moreover, the AMPKα inhibitor, compound C, specifically inhibited AYN‐induced GLUT4 expression in 3T3‐L1 adipocytes (Figure 6D), concurrently suppressing glucose uptake (Figure 6E). These findings elucidate that AYN can activate the AMPKα/GLUT4 signaling pathway at the cellular level in vitro, thereby enhancing cellular glucose uptake and potentially contributing to the reduction of blood glucose levels in diabetic mice.

### 3.6 AYN Reduced Macrophage Infiltration and Inflammatory Activation in epWAT

**FIGURE 7:**
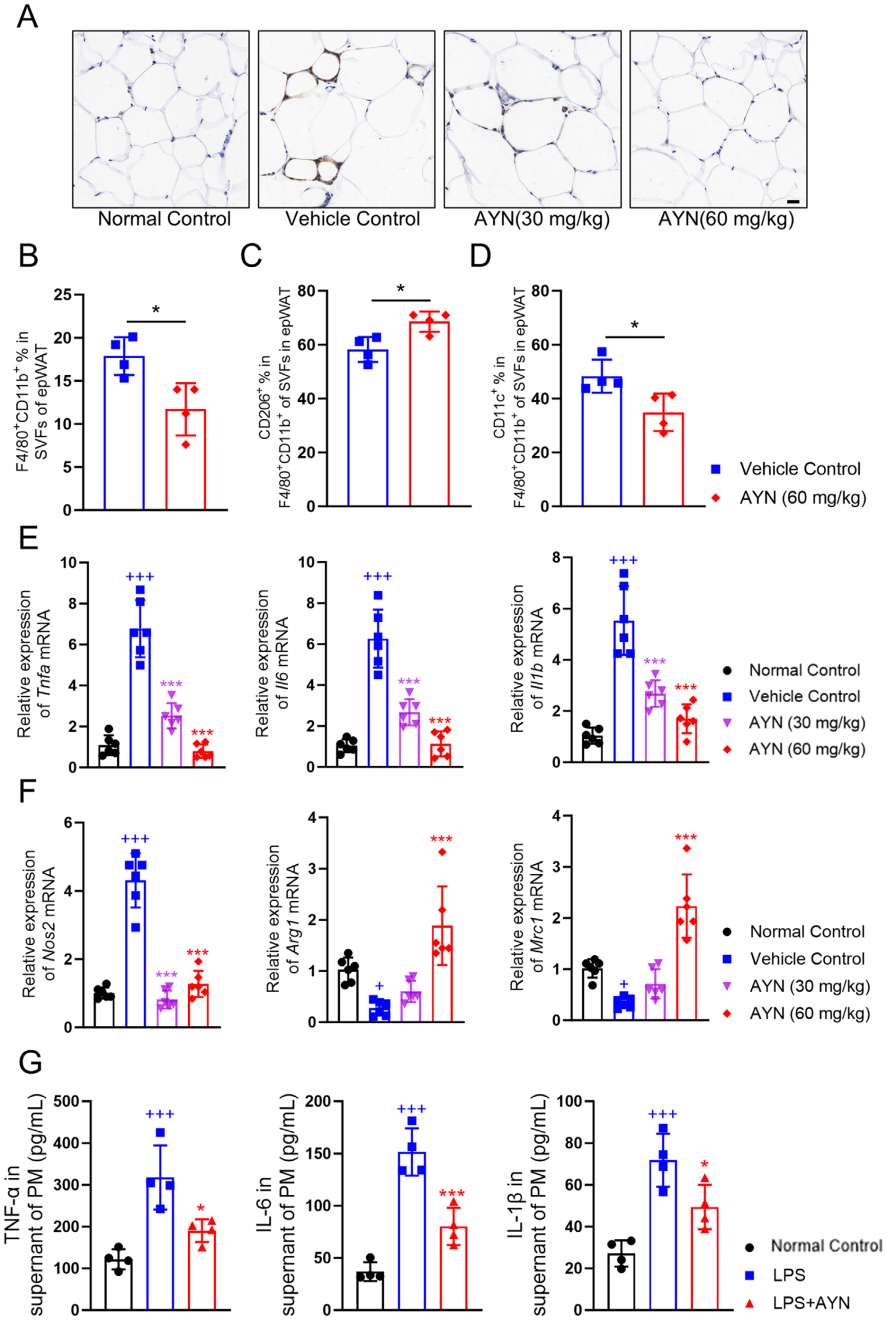
AYN reduced macrophage infiltration and inflammatory activation. (A) F4/80^+^ immunohistochemistry staining for epWAT; (B) F4/80^+^CD11b^+^ macrophages proportion in SVFs of epWAT, (C) CD206^+^ macrophages proportion in F4/80^+^CD11b^+^ macrophages, and (D) CD11c^+^ macrophages proportion in F4/80^+^CD11b^+^ macrophages. (E) AYN decreased *Tnfa* (Left), *Il6* (Middle) and *Il1b* (Right) mRNA expression in epWAT; (F) AYN decreased *Nos2* (Left), but increased *Arg1* (Middle) and *Mrc1* (Right) mRNA expression in epWAT; (G) AYN decreased TNF‐α (Left), IL‐6 (Middle) and IL‐1β (Right) secretion induced by LPS in supernatant of peritoneal macrophages (*n* = 3–6, *p < 0.05, **p < 0.01, ***p < 0.001, compared with vehicle control group or LPS group in G; ^+^ *p* < 0.05, ^+++^ *p* < 0.001, compared with Normal control group).

It is widely recognized that chronic inflammation within obese adipose tissue is a key contributor to the development of insulin resistance (Lee and Olefsky 2021). Macrophages, which are the predominant immune cells in adipose tissue, play a critical role in regulating the immune microenvironment through alterations in their phenotypes and functions (Olefsky and Glass 2010). Suppressing the pro‐inflammatory activation and reducing the infiltration of macrophages in obese adipose tissue can alleviate inflammation and subsequently enhance insulin sensitivity by increasing GLUT4 expression (Liang et al. 2015; Wang et al. 2025). In the epWAT of the vehicle group, F4/80^+^ macrophages were abundantly infiltrated and formed characteristic “crown‐like structures” around adipocytes, indicating significant inflammation in epWAT. Administration of AYN reduced the infiltration of F4/80^+^ macrophages in epWAT (Figure 7A). The flow cytometry analysis of SVFs in epWATs revealed a reduction in the proportion of F4/80^+^CD11b^+^ macrophages in the epWAT of the group treated with 60 mg/kg AYN compared to the vehicle group (Figure 7B). Subsequent phenotypic characterization of F4/80^+^CD11b^+^ macrophages demonstrated a decrease in the proportion of CD11c^+^ macrophages (Figure 7D), indicative of a pro‐inflammatory phenotype, while the proportion of CD206^+^ macrophages (Figure 7C), associated with an anti‐inflammatory phenotype, showed a slight increase following the 60 mg/kg AYN treatment (Chen et al. 2021). These findings suggest that AYN may modulate macrophage activation towards a less pro‐inflammatory state, potentially ameliorating glucose and lipid dysregulation in epWAT. To further elucidate the anti‐inflammatory effects of AYN in epWAT, quantitative PCR (qPCR) was employed to assess the mRNA expression levels of pro‐inflammatory markers (*Tnfa*, *Il6*, *Il1b*, and *Nos2*) and anti‐inflammatory markers (*Arg1* and *Mrc1*). The AYN treatment resulted in a downregulation of pro‐inflammatory genes and an upregulation of anti‐inflammatory genes (Figure 7E,F). In vitro experiments corroborated these findings, demonstrating that AYN inhibited the secretion of TNF‐α, IL‐6, and IL‐1β by LPS‐stimulated peritoneal macrophages (Figure 7G). These results indicated that AYN may ameliorate the lipid and glucose dysfunction partly by inhibiting the inflammatory activation of macrophages in epWAT.

## 4 Discussion

Type 2 diabetes mellitus (T2DM) is characterized by reduced insulin sensitivity and chronic systemic inflammation in metabolic tissues, including adipose tissue, skeletal muscle, and the liver (Esposito et al. 2024; Hill et al. 2021; Mulvihill et al. 2011). GLUT4, a critical transporter responsible for mediating glucose uptake in adipocytes and skeletal muscle cells, has been extensively documented to exhibit diminished expression in adipose tissue and skeletal muscles during the progression of insulin resistance (Lv et al. 2021; Wu et al. 2024; Zhou et al. 2023). The suppression of GLUT4, induced by prolonged exposure to elevated glucose and lipid levels as well as inflammatory activation in tissues, impedes the entry of glucose into cells for metabolism and utilization. This exacerbates the metabolic dysfunction of glucose and lipids, thereby further deteriorating the condition of T2DM (Leto and Saltiel 2012). Therefore, compounds derived from edible natural products that directly upregulate GLUT4 expression, ameliorate glucose and lipid metabolic dysfunction, and concurrently reduce tissue inflammation possess significant therapeutic potential for alleviating symptoms of T2DM (Lv et al. 2019, 2020; Qi et al. 2025). Flavonoids, abundant in various edible natural plants, have demonstrated potential effects in lowering blood glucose levels, regulating dyslipidemia, and ameliorating inflammation in metabolic tissues, suggesting that flavonoids may serve as dietary supplements for diabetes management. Notably, representative flavonoids such as quercetin, acacetin, and baicalein have been identified to directly target GLUT4 expression, thereby ameliorating insulin resistance (Kwon et al. 2020; Min et al. 2018; Neisy et al. 2019).

AYN (Ayanin) is a representative flavonoid compound found in edible natural products (Yu et al. 2024), and there is a lack of studies investigating the potential of AYN in increasing GLUT4 expression, ameliorating inflammation and further improving T2DM. In this study, T2DM was experimentally induced in mice through extended HFD feeding combined with STZ injections. These mice are well‐established models for T2DM, characterized by hyperglycemia, lipid metabolism disorders, chronic inflammation and relatively insufficient insulin secretion (Lv et al. 2021). Subsequently, oral administration of AYN was employed to examine its potential anti‐diabetic effects. Our findings indicate that oral administration of AYN significantly reduced blood glucose levels in these mice and improved both OGTT and insulin sensitivity. Furthermore, AYN treatment inhibited insulin secretion, likely as a consequence of the observed improvements in glucose and insulin tolerance. The aforementioned results preliminarily suggest that AYN administration may have the potential to mitigate hyperglycemia and insulin resistance.

Obesity is a significant factor contributing to the development of T2DM, with prolonged obesity being associated with an elevated risk of insulin resistance and chronic inflammation (Boutari et al. 2023; Zhu et al. 2021). Weight reduction in diabetic patients can mitigate or even reverse the progression of T2DM (Sandoval and Patti 2023). Oral administration of AYN has been shown to significantly decrease body weight in diabetic mice, suggesting that AYN may ameliorate diabetic symptoms by targeting this critical condition for T2DM onset. Adipose tissue is a crucial metabolic organ, primarily regulating energy metabolism through the lipogenic and lipolytic activities of adipocytes (Ahmed et al. 2021). Excessive lipid accumulation in adipocytes significantly contributes to overweight, chronic tissue inflammation, and insulin resistance (Ahmed et al. 2021; Jeon et al. 2023). Agents that ameliorate hypertrophic adipocytes, inhibit inflammation, and improve dysfunction in adipose tissues may aid in enhancing insulin resistance and managing T2DM (Lv et al. 2019; Xia et al. 2024). AYN has been observed to improve adipocyte hypertrophy and decrease F4/80^+^ macrophages infiltration in adipose tissues of diabetic mice, indicating that AYN may enhance insulin sensitivity and ameliorate diabetic symptoms by modulating lipid storage and ameliorating inflammation in adipose tissues.

Diabetes is commonly linked to dyslipidemia, a condition marked by increased concentrations of LDL‐C, TG, TC, and FFA in both serum and tissues, as well as reduced levels of HDL‐C (Gao et al. 2025; Kaze et al. 2021; Mulvihill et al. 2011). Numerous studies have demonstrated that dyslipidemia in serum and metabolic tissues, such as skeletal muscle, adipose tissue, and liver, is a significant factor contributing to impaired insulin sensitivity, ultimately leading to hyperglycemia (Gross et al. 2017; Jiang et al. 2024; Mulvihill et al. 2011; Qi et al. 2025). In the present study, the administration of AYN led to a significant reduction in the concentrations of TG, TC, and FFA in serum, skeletal muscle, and liver, while also promoting a decrease in serum LDL‐C levels and an increase in HDL‐C levels. The findings indicate that AYN may have the potential to alleviate dyslipidemia associated with T2DM, thereby contributing to the improvement of insulin sensitivity and the reduction of blood glucose levels.

AMP‐activated protein kinase (AMPK) is widely acknowledged as a critical regulator of cellular energy status (Herzig and Shaw 2018). Upon activation via phosphorylation, phosphorylated AMPKα (p‐AMPKα) augments the expression and translocation of GLUT4, thereby promoting glucose uptake and transport in an insulin‐independent manner (Wu et al. 2024). Agents that specifically activate p‐AMPKα may exert beneficial effects on the regulation of GLUT4 expression in metabolic tissues (Zhou et al. 2023). In this study, we observed elevated expression levels of GLUT4 in the skeletal muscle and epWAT of mice treated with AYN, with the expression pattern of p‐AMPKα mirroring that of GLUT4. This suggests that the increased expression of GLUT4 in these tissues may be modulated by elevated p‐AMPKα levels. Subsequent cellular analyses revealed that AYN treatment led to a significant, dose‐dependent upregulation of GLUT4 and p‐AMPKα expression in 3T3‐L1 adipocytes, concomitant with an enhancement in glucose uptake. Furthermore, the application of the AMPKα activation inhibitor, Compound C, markedly reduced GLUT4 expression in 3T3‐L1 adipocytes and inhibited their glucose uptake. The findings indicate that AYN has the potential to activate the AMPKα/GLUT4 signaling pathway in both in vivo and in vitro settings, thereby ameliorating hyperglycemia associated with diabetes. Moreover, the activation of AMPKα may upregulate the expression of the CPT‐1α protein. This protein is essential for the conjugation of long‐chain fatty acids with carnitine, thereby facilitating their translocation across the mitochondrial membrane—a rate‐limiting step in cellular fatty acid β‐oxidation (Dong et al. 2024). The upregulation of CPT‐1α expression can facilitate lipid metabolism in adipocytes, hepatocytes, and skeletal muscle cells, thereby mitigating the lipid metabolic disorders associated with T2DM (Dong et al. 2024). AYN has been shown to enhance CPT‐1α expression in the adipose tissue, skeletal muscle, and liver of diabetic mice, with the expression pattern of CPT‐1α aligning with that of p‐AMPKα. This suggests that the AYN‐induced upregulation of CPT‐1α may be modulated by p‐AMPKα. Consequently, we propose that AYN ameliorates the lipid metabolic disorders associated with T2DM through the activation of the AMPKα/CPT‐1α signaling pathway.

The inflammatory microenvironment within epWAT suppresses GLUT4 expression and exacerbates insulin resistance, with macrophages playing a pivotal role as key immune cells in regulating this microenvironment (Moraes‐Vieira et al. 2016). Pro‐inflammatory macrophages, characterized by elevated expression of CD11c^+^ and *Nos2*, secrete inflammatory cytokines such as TNF‐α, IL‐6, and IL‐1β, thereby inhibiting GLUT4 expression and worsening insulin resistance (Chen et al. 2021). Conversely, anti‐inflammatory macrophages, identified by increased expression of CD206^+^ and *Arg1*, mitigate insulin resistance and enhance GLUT4 expression by clearing apoptotic adipocytes and secreting anti‐inflammatory cytokines (Chen et al. 2021). Research has demonstrated that agents capable of inhibiting pro‐inflammatory activation while simultaneously promoting anti‐inflammatory activation of macrophages in epWAT may enhance GLUT4 expression in these tissues, thereby potentially ameliorating insulin resistance (Olefsky and Glass 2010). Our findings demonstrate that AYN intervention led to a reduction in the infiltration of F4/80^+^ macrophages, particularly CD11c^+^ macrophages, while slightly increasing the presence of CD206^+^ macrophages. This was accompanied by the inhibition of pro‐inflammatory gene expression and the promotion of anti‐inflammatory gene expression. Furthermore, in vitro experiments revealed that AYN inhibited the secretion of pro‐inflammatory cytokines induced by LPS in peritoneal macrophages. The findings indicate that the anti‐inflammatory properties of AYN may play a role in mitigating hyperglycemia and insulin resistance.

In conclusion, this study is the first to demonstrate, using in vitro cell models and HFD feeding in conjunction with STZ‐induced T2DM mouse models, that the flavonoid compound AYN alleviates symptoms associated with T2DM. This effect is mediated through the upregulation of GLUT4 and CPT‐1α expression via the activation of AMPKα. Additionally, the reduction of macrophage‐mediated chronic inflammation in epWAT partially contributes to the upregulation of GLUT4 expression and the amelioration of insulin resistance.

## 5 Conclusion

Ayanin, a flavonoid commonly found in edible plants, has demonstrated potential anti‐diabetic effects. In vivo studies have shown that intervention with AYN reduces hyperglycemia, improves oral glucose tolerance and insulin tolerance tests, alleviates inflammation in adipose tissues, and mitigates dyslipidemia in diabetic mouse models. These beneficial effects are likely due to the activation of the AMPKα/GLUT4 and AMPKα/CPT‐1α pathways, as well as the anti‐inflammatory activity of AYN. In vitro experiments revealed that AYN enhances glucose uptake in 3T3‐L1 adipocytes by specifically activating AMPKα, which subsequently increases GLUT4 expression. Additionally, AYN inhibits the secretion of pro‐inflammatory cytokines in LPS‐activated peritoneal macrophages. Overall, AYN emerges as a promising anti‐diabetic agent by promoting GLUT4 expression and reducing tissue inflammation.

## Author Contributions

Writing original draft, conceptualization, investigation: Y.L., C.W., Z.G. and J.H. Table and figure generation: W.Y., X.X, D.W., K.Y., and J.W. Critical revision: S.L., X.X. and Z.G. All authors have read and agreed to the published version of the manuscript.

## Funding

The work in this paper was supported by the Natural Science Foundation of China (Grant No. 82371864), the Natural Science Foundation of Henan Province (Grant No. 232300421121), and the Major Projects Jointly Constructed by Henan Province and the Ministry of Science and Technology (Grant No. SBGJ202101003).

## Ethics Statement

Ethical approval for the mice‐related experiments in this study was granted by Zhengzhou University Research Ethics Committee, Reference number ZZUAFD2025‐0089.

## Conflicts of Interest

The authors declare no conflicts of interest.

## Supporting information


**Data S1:** fsn371429‐sup‐0001‐Supinfo.docx.

## Data Availability

The data that support the findings of this study are available from the corresponding author upon reasonable request.
