# Supplementary material for: Anti‐Diabetic Effects of Ayanin, a Flavonoid Compound, in STZ/HFD‐Induced Diabetic Mice by Upregulating GLUT4 and Suppressing Macrophage‐Driven Inflammation in Adipose Tissues
Source: Food Sci Nutr. 2026 Jan 7;14(1):e71429. doi: 10.1002/fsn3.71429 (PMC12778411; doi:10.1002/fsn3.71429)
Supplement: Supplementary file 1 — Data S1: fsn371429‐sup‐0001‐Supinfo.docx. [file FSN3-14-e71429-s001.docx]

Primer sequence:

m *Tnfa* Forward: GACGTGGAACTGGCAGAAGAG

m *Tnfa* Reverse: TTGGTGGTTTGTGAGTGTGAG

m *Il6* Forward: TAGTCCTTCCTACCCCAATTTCC

m *Il6* Reverse: TTGGTCCTTAGCCACTCCTTC

m *Il1b* Forward: GCAACTGTTCCTGAACTCAACT

m *Il1b* Reverse: ATCTTTTGGGGTCCGTCAACT

m *Nos2* Forward: GTTCTCAGCCCAACAATACAAGA

m *Nos2* Reverse: GTGGACGGGTCGATGTCAC

m *Arg1* Forward: CTCCAAGCCAAAGTCCTTAGAG

m *Arg1* Reverse: AGGAGCTGTCATTAGGGACATC

m *Mrc1* Forward: CTCTGTTCAGCTATTGGACGC

m *Mrc1* Reverse: CGGAATTTCTGGGATTCAGCTTC

m *Actb* Forward: GGCTGTATTCCCCTCCATCG

m *Actb* Reverse: CCAGTTGGTAACAATGCCATGT
